# Let’s Talk aBOT Scam Online Survey Completions in Health Behavior Research: Tutorial With Case Studies, Practical Guidance, and a Checklist for Researchers

**DOI:** 10.2196/76622

**Published:** 2025-12-24

**Authors:** Lauren Arundell, Jo Salmon, Anthony Walsh, Kathleen Dullaghan, Heilok Cheng, Thea Baker, Megan Teychenne, Felipe Schuch, Debora Tornquist, Anna Timperio

**Affiliations:** 1 Institute for Physical Activity and Nutrition (IPAN) Deakin University Burwood, Victoria Australia; 2 Department of Sports Methods and Techniques Universidade Federal de Santa Maria Santa Maria Brazil; 3 Faculty of Health Sciences Universidad Autónoma de Chile Providêcia Chile; 4 Institute of Psychiatry Universidade Federal do Rio de Janeiro Rio de Janeiro Brazil; 5 Graduate Program in Movement Sciences and Rehabilitation Universidade Federal de Santa Maria Santa Maria Brazil

**Keywords:** scam, bots, survey, online, data integrity, data fabrication, falsification

## Abstract

**Background:**

Online data collection can reach large populations efficiently and cost-effectively. However, the increase in bots and scammers (ie, person- or software-based fraudulent completions) completing online surveys raises data integrity issues and wastes scarce research resources.

**Objective:**

This paper aims to describe case studies and experiences in which bot or scam completions of online surveys occurred within the health behavior field (specifically physical activity and nutrition). Lessons learned and a checklist of strategies to assist researchers before, during, and after data collection to reduce the incidence of and identify bot or scam completions are provided.

**Methods:**

Four diverse case studies are presented from studies that used online recruitment and data collection methods for cross-sectional surveys by parents about children’s screen time, cross-sectional surveys by adults about transport-related physical activity, qualitative interviews for a proposed trauma-informed physical activity program for female victim-survivors of intimate partner violence, and the Australian component of a large multicountry prospective study targeting university students. The strategies used to identify and prevent bot or scam online survey completions are explored.

**Results:**

High levels (7%-80%) of suspected bot or scam completions were identified in a number of these studies. Participant characteristics and outcome variables were significantly different between included and excluded participants (eg, excluded responses had a higher percentage of male parents and children, higher social media use, and lower physical activity guideline adherence). The learnings from these case studies and the wider literature are combined to create a checklist of strategies that researchers can use to prevent and identify bot or scam completions. These include strategies before data collection (when creating study collateral), during survey design and development (including the use of inbuilt platform functions and the design of the survey questions and structure), following data collection (indicators of potential bot or scam completions), and recommendations for reporting of bots or scams.

**Conclusions:**

The checklist, based on the included case studies and wider literature, can be used to help researchers who use online recruitment and data collection methods at each stage, from planning and conducting through to analyzing and reporting their findings. Researchers should include several steps to prevent and identify fraudulent survey responses when creating surveys and completing data cleaning. This checklist should also be considered in grant applications and ethics applications. This will provide greater confidence in the research findings and reduce unnecessary waste of research time and resources.

## Introduction

Online data collection is an efficient and cost-effective method to reach a wide audience [1]. However, reports of online surveys being completed by bots (software programs that can complete repeated tasks at speed [2]); by individuals fraudulently claiming to be eligible participants (scammers); and by people completing the survey with malicious intent to sabotage the project or system (bad actors) are becoming widespread and can lead to economic loss, loss of trust, and identity theft [3,4]. In research [2,5-7], these practices raise data integrity concerns, impact the ability to draw accurate conclusions or take appropriate actions based on survey findings, and strain scarce research resources, especially when an incentive (eg, a voucher) is offered for completion. While most survey platforms provide some bot or scam prevention methods [8], these alone may be insufficient [5], and researchers using online methods need practical recommendations to ensure the rigor of their data [9]. The purpose of this paper is to describe case studies and experiences of potential bot or scam completions of online surveys (for either recruitment or data collection purposes) within the health behavior field, specifically physical activity and nutrition; and to provide a checklist of practical strategies for researchers to help reduce and identify bot or scam completions before, during, and after data collection, informed by these case studies and the wider literature [2,5,6,10]. The checklist may also support submissions for journals, grant agencies, and ethics committees so that publications and research funders can take precautions to avoid bot or scam completions.

## Ethical Considerations

Ethics approval for the included case studies was obtained from the following institutes: case study 1, ethics approval granted from Deakin University Human Research Ethics–Health (HEAG-H 103_2024); case study 2, ethics approval granted from Deakin University Human Research Ethics (2024/HE000064); case study 3, ethics approval granted from Deakin University Human Research Ethics (2024-209); and case study 4, ethics approval granted from Deakin University Human Research Ethics (2022-311) and Brazil (CAE:63025822.8.1001.5346). Participants were provided with a digital plain language statement outlining the study requirements, privacy and confidentiality of their data and any compensation provided (specific to the case study). Informed consent (digital) was then obtained from participants.

## Recent Case Studies

We present 4 recent case studies of surveys conducted in the health (specifically physical activity and nutrition) research field in Australia where bot or scam completions were detected (Textboxes 1-4). Three case studies offered a supermarket voucher to each participant, and 1 case study entered participants into an e-gift card draw. The estimated percentage of nonlegitimate participants and differences in results between the included and excluded participants (where available) are presented for each case study in Textboxes 1-4.

**Case study 1: online recruitment of parents to complete a survey about screen time (Australia).**

**Population target**

- Parents of children aged 8-16 years in Australia.

**Recruitment method**

- Recruitment was conducted through social media platforms.

**Survey platform**

- The survey was hosted on Qualtrics (Qualtrics, LLC)

**Compensation provided**

- Participants received an AUD $10 (US $6.70) supermarket e-voucher

**Suspected nonlegitimate completions**

- 80% of the responses were identified as nonlegitimate.

**Study procedure**

Social media (Facebook and Instagram) recruitment for an online survey using Qualtrics commenced in June 2024. Participants completed an initial screening questionnaire, and eligible participants (ie, a parent of a child aged 8 to 16 years living in Australia) provided consent (via online checkbox), then progressed to complete the full survey. Survey completers would receive an AUD $10 (US $6.70) supermarket e-voucher. The survey was closed after 5 days as more than 600 surveys had been completed.

**Steps to identify potentially fraudulent responses**

The research team examined the Qualtrics embedded data (eg, bot detection variable) and identified mismatched responses (eg, a mismatch between state and postcode) and unlikely responses (eg, a very high number of children, identical or similar dates of birth completed largely sequentially). Emails were sent to participants requesting verification of responses identified as dubious. Suspicious replies were received with standardized responses, font, and format, questionable email addresses (eg, mix of random letters and numbers), and multiple emails from the same “parent” email address. Before reopening the survey for further participant recruitment, additional prevention steps were added, including a reCAPTCHA survey item, a “honeypot” question (a nonsense question that is hidden to people but completed by bots), additional cross-checking questions (eg, participant date of birth check at the start and end of the survey), and specifications that the e-vouchers were limited to an Australian store and that nonlegitimate responses would not receive an e-voucher.

**Results**

The final sample was 1248 responses, of which 995 (80%) were identified as potentially nonlegitimate and excluded. Including the additional prevention checks slightly improved the proportion of legitimate completions from 16% (before the additional prevention steps) to 25% (after additional prevention checks). There were significant differences in results between the included and excluded participants including the sex of parents (male participant: 37% vs 53%, *P*<.01) and children (male participant: 53% vs children: 60%, *P*<.042), child age at first mobile phone ownership (9.9 vs 8 years, *P*<.001), minutes of social media use per weekday (4 h 19 min/day vs 5 h 44 min/day, *P*<.030), and days per week of meeting physical activity guidelines (3 vs 2.6 days/week, *P*<.017).

**Case study 2: online recruitment for a Brisbane transport and physical activity survey (Australia).**

**Population target**

- Adults aged 18-64 years living in Greater Brisbane, Australia

**Recruitment method**

- Recruitment was conducted through Facebook advertisements.

**Survey platform**

- The survey was administered using Qualtrics.

**Compensation provided**

- Participants received an AUD $10 (US $6.70) supermarket e-voucher.

**Suspected nonlegitimate completions**

- 59% of the responses were identified as nonlegitimate.

**Study procedure**

A Facebook campaign recruited Greater Brisbane residents (aged 18-64 years) for an online survey on transport-related physical activity in July 2024. The target was 450 participants within 2 weeks. Survey completers were offered an AUD $10 (US $6.70) supermarket e-voucher. Participants first completed a screening questionnaire to confirm eligibility, provided informed consent (via an online checkbox), and submitted contact details (first name, email, and mobile number) along with the approximate residential address (local government area, postcode, suburb, and nearest cross-street). The campaign generated 1311 complete and 652 incomplete screening responses.

**Steps to identify potentially fraudulent responses**

The research team used Qualtrics’ built-in tools and manual checks based on a combination of the following: mismatched or implausible location data (eg, outside Brisbane, incorrect suburb-postcode pairs, and missing location data); reCAPTCHA issues (eg, low scores or repeated failed attempts); suspicious email addresses (eg, random characters) or landline numbers instead of mobile or cellphone; identical or nearly identical address details submitted consecutively; providing a first name and surname instead of just a first name.

**Results**

A total of 767 (59%) responses were excluded as possibly fraudulent, mostly due to having a combination of issues identified using the Qualtrics tools and manual checks. This left 544 participants considered probably legitimate, who were then invited to complete the full survey.

**Case study 3: online recruitment for a qualitative study of victim-survivors of intimate partner violence (IPV) about a noncontact cardio-boxing program (Australia).**

**Population target**

- Women victim-survivors of any form of IPV who were aged 18 years or older and living in Australia

**Recruitment method**

- Recruitment was conducted through social media platforms.

**Survey platform**

- The survey was hosted on Qualtrics.

**Compensation provided**

- Participants received an AUD $30 (US $20.10) supermarket e-voucher.

**Suspected nonlegitimate completions**

- 16% of the responses were identified as nonlegitimate.

**Study procedure**

Social media platforms (Facebook and Instagram) were used to recruit participants for a brief online interview exploring perceptions of a proposed noncontact cardio-boxing program for women who have experienced IPV. Recruitment began in November 2024.

After watching a brief introductory video on the research project, respondents were asked to complete an initial screening questionnaire. Eligible participants (ie, women with a lived experience of any form of IPV, living in Australia, and older than 18 years with a secure internet connection) then provide consent (via online checkbox) and preferred contact details (ie, text, email, or phone call) to arrange for an online (video) interview. Interview completers would be offered an AUD $30 (US $20.10) supermarket e-voucher. Within 11 days, 25 participants met the inclusion criteria and provided contact information to take part in an interview.

**Steps to identify potentially fraudulent responses**

The steps included using the bot detection and reCAPTCHA tools within Qualtrics, manually matching postcode to state and cross-referencing those to the GeoIP Estimation data, and identifying suspicious email addresses (ie, combinations of names and numbers) and timing of survey completion.

**Results**

Following analysis, of the 25 responses, 4 (16%) were deemed to be scam responses, while a further 6 (24%) participants flagged by the system as potential bots proved to be legitimate participants.

**Case study 4: a large multicountry prospective study targeting university students.**

**Population target**

- Undergraduate university students worldwide

**Recruitment method**

- Recruitment was conducted through university course websites (posted by course or unit coordinators), flyers, and social media platforms.

**Survey platform**

- The survey was administered using REDCap (Research Electronic Data Capture).

**Compensation provided**

- Participants were entered into a prize draw to win AUD $100 (US $67) e-gift cards at each data collection time point (Australian cohort).

**Suspected nonlegitimate**

- 7% of the Australian sample was identified as nonlegitimate (international data collection ongoing).

**Study procedure**

This large prospective study, involving data collection via online surveys at 4 time points (across 3.5 years), recruited undergraduate university students from 84 universities across 27 countries (worldwide). A wide recruitment campaign for the Australian sample included advertising the study to students at participating universities via course or unit (websites, displaying flyers around the university, and nonpaid social media dissemination [X and Instagram]). Recruitment began at the first study site (university) in Australia in February 2023 (for a duration of 1 teaching semester or trimester). A second wave of recruitment was undertaken the following year (February 2024) to boost the sample size at 5 out of 6 universities. Interested participants completed a brief screening survey to assess eligibility (ie, undergraduate university student, studying in their first year, and at a participating university). Within 4 weeks, a total of 631 participants in the Australian cohort met the inclusion criteria and started or completed the online surveys. However, of these, 111 (17.6%) were suspected bots, scams, or bad actors.

**Steps to identify potentially fraudulent responses**

The steps included identifying reported fake email addresses; answers in non-English languages; and clearly fake responses (eg, last university enrollment at ≥40 years ago, reporting a net income of over AUD $1 million per month, self-reported occupation as “Microsoft owners,” and self-reported weight and height with unrealistic values).

To address this initial bot attack, additional preventive measures were put in place, including ensuring re-CAPTCHA tools were activated and optimized within the REDCap screening survey, as well as subsequent data collection surveys.

The use of X (formerly Twitter) for recruitment was also paused, because it was suspected that this platform allowed broader (less targeted or more widespread) public access to the survey compared with other recruitment strategies.

**Results**

Overall, for the Australian cohort, a total of 2869 participants completed the baseline survey; however, 189 (6.9%) were suspected bots. For the global sample (data collection still ongoing), a total of 35,638 (partial) participants have completed the baseline survey, with 178 (0.5%) suspected bots. Because the Australian sites were the first of the global cohort to start recruitment, their learnings were able to be used by the global research team to help prevent or manage bot attacks during recruitment for subsequent sites and countries.

## Considerations and Checks to Reduce the Incidence of and Identify Bot or Scam Completions

Based on the learnings from these case studies and the wider literature [2,5,6,10], the following considerations and checks have been compiled to assist researchers when designing and conducting their research to help reduce the incidence of, and identify bot or scam completions. These have been developed into a checklist shown in Multimedia Appendix 1.

Some considerations or checks may be sufficient on their own to suggest a nonlegitimate completion (eg, when the honeypot question is completed), whereas a combination of different checks may be needed to identify a potential nonlegitimate completion (eg, a run of similar start times, *plus* a questionable email address, *plus* similar responses to open-ended questions) to avoid the exclusion of genuine respondents. The following considerations and checklist can be used by researchers to develop a study-specific list of considerations and checks, including hierarchies and combinations of criteria, which can then be applied to each phase of their study to help identify potentially nonlegitimate completions.

### Before Data Collection: Creating Study Collateral

Information can be included in the plain language statements, consent forms, and advertisements to help deter bots or scams and indicate how potentially fraudulent data will be dealt with. For example, researchers can reduce the prominence of advertising vouchers or payment as compensation, specify the type of voucher and where it can be redeemed (eg, for a country-specific retailer), state that completions are limited to 1 per household, notify participants that they may be asked to confirm their identity via their listed contact method (eg, email or phone number), and indicate that suspected fraudulent completions will not receive the voucher.

### Survey Design and Development 1: Built-In Survey Platform Functions

When creating the survey, enable the built-in bot or scam detection functions of the survey platform. These tools may automatically navigate respondents out of the survey if deemed to be fraudulent (bot or scam) or add a variable to the collected data that can be later reviewed and interpreted as potentially problematic.

In Qualtrics [8], *Bot detection* provides a *Q_RecaptchaScore* (a score of <0.5 is likely to be a bot); and *Prevent multiple submissions* adds a *Q_BallotBoxStuffing* value (a score of 1 is likely to be a duplicate) for each completion. Enabling the *Relevant ID* functions (*Duplicate*, *Duplicate Score*, *Fraud Score*, and *Last Start Date*) helps detect multiple responses by looking at respondents’ metadata. The *Duplicate* field will show “True” with the corresponding date and time the last attempt was started in the *Last Start Date* field. *Duplicate Scores* of ≥75 indicate duplicate responses, while *Fraud Scores* of ≥30 indicate the response is both fraudulent and likely a bot. The reCAPTCHA feature, where the participant needs to correctly click the squares containing an item (eg, motorcycles), can also be added to a survey.

In REDCap (Research Electronic Data Capture), reCAPTCHA is also available [11]. Similarly, a similar IP address or geolocation tracking is available through 3 options: set-up of consent forms as e-consent forms, where the IP address is automatically recorded; use of geolocation action tags in survey fields, to identify the longitude and latitude of the survey respondent; and external modules that identify IP address [12]. However, each option has limitations: reCAPTCHA may be passed by sophisticated bots; IP addresses may be falsified with virtual private network services or proxy servers; use of geolocation tags may be blocked by respondents’ browsers; and use of external modules is determined by individual REDCap server managers [13].

### Survey Design and Development 2: Online Survey Question and Structure

Several precautions can be added to surveys during the design phase to exclude or identify bot or scam completions. These may include the following measures:

- Including a reCAPTCHA variable
- Applying branching logic to exclude ineligible responses from progressing, ensuring that a participant must correctly answer the eligibility question to continue
- Using open-ended questions which provides an additional check as they must type the correct response
- Adopting a 2-step process separating screening and consent form completion from the study survey (eg, eligible participants are sent a unique link to the full survey via their email address, only after responses to the screening questions have been assessed by both the survey platform functions and the researcher)
- Adding automated date and time survey fields, which can be coded to be hidden from respondents (eg, action tags in REDCap can record the respondent’s current date and time based on the host REDCap server, and current date and time based on Coordinated Universal Time); discrepancies in timestamps can indicate responses from outside the target region or time zone.
- Repeating questions where the response would not typically change to enable cross-checking (eg, name, date of birth, email, and postcode, in separate consent and survey sections)
- Adding dummy “honeypot” questions (eg, “Please select your favorite option: 1, 2, or 3”), coded to be hidden to humans and only visible to bots. Completion of the question indicates the respondent is nonlegitimate
- Including attention check questions to progress (eg, “Select a color from this list to continue,” response options: cat, blue, broccoli)
- Requesting additional contact information to verify participant eligibility

### Following Data Collection: Identifying Indicators of Potential Bot or Scam Completions

Researchers should promptly and continuously review their data to identify potential bot or scam completions. The following are factors based on embedded data (eg, IP address), participant responses, and response patterns, which can help identify potentially fraudulent responses:

- Completed responses to the honeypot question (ie, competed by a bot).
- Incorrect geolocation for the study sample (eg, latitude or longitude out of range).
- Built-in bot detection function or variables (eg, Qualtrics *Q_RecaptchaScore*<0.5, *Duplicate Score* of ≥75, *Fraud Score* of ≥30).
- Duplicate IP addresses: however, this may not be a reliable indicator where participants could be at the same location (eg, workplace, school, public library, or internet café) or where multiple responses are permitted from the same address or family or household.
- Similar IP addresses (ie, all but the final sequence of numbers are identical) may indicate repeated submissions from similar sources.
- Several failed attempts at passing a reCAPTCHA test, followed by a successful completion from the same IP address; however, this requires the researcher to examine and compare incomplete and complete surveys.
- Unrealistic survey completion time (eg, less than 10 min when the median time was 33 min).
- Multiple consecutive responses with similar survey start and end time, or in a run of start and end times (eg, a survey with a median completion length of 30 min starts at 5:00 PM and takes 7 min to complete; another survey starts at 5:07 PM, and so on).
- Multiple participants with the same personal details (eg, first name and surname, email address, phone or mobile number, home address).
- Questionable email address or names, often with a similar pattern and completed at a similar time (eg, a participant called Jane Doe provides an email address of john.smith123@...; a run of similar name formats, such as, including a middle initial or extra spaces between names; email addresses contain a random mix of letters and numbers with similar pattern between participants, such as bgckts541236@... or 74sqwpc.hx54612@...).
- Unusual responses for study context (eg, in studies recruiting parents or perinatal women, this may include an unusually high number of children, particularly over a short or unrealistic time frame, or unrealistic age of childbirth, such as more than 9 children from live births, child birth dates only 6 months apart, child birth at parent age less than 12 years).
- Mismatch between data provided at 2 locations of the participation process (eg, mismatch between date of birth reported in the consent form and survey or state and postcode provided do not match).
- Identical or very similar responses to an open-ended question. This should not be applied to likely common responses (eg, “none,” “no benefits,” “schoolwork,” etc).
- Questionable date format used for date-based responses (eg, including full stops when backslashes are requested or writing the month instead of the number format).
- Providing a landline phone number when a mobile number is requested, or providing landline phone numbers that do not match the area or city they are claiming to live in.
- Providing a full name when only a first name was requested.
- Nonresponse to requested contact information, which would allow researchers to check and confirm details.
- Completed at unusual or unlikely times, such as between 12 AM and 5 AM. Use participant-specific context as needed (eg, consider postnatal women with disrupted sleep patterns, or night shift workers).

### Analysis and Write-Up: Reporting of Potential Bot or Scam Completions

Researchers should include a description of any suspected bot or scam completions in reporting their findings using the checklist in Multimedia Appendix 1. This should include how they were identified, the number identified, and reporting confirmation that they were excluded. This provides greater confidence that the participants included and the subsequent findings of the study are legitimate.

### Additional Considerations

It is important to note that with increasingly sophisticated bots or scams, not all potential bot or scam completions may be identified, and some legitimate completions may be incorrectly excluded (eg, case study 3). These preventive measures may also inadvertently contribute to the exclusion of some groups, for example, those with low literacy or digital skills. As the technology landscape is rapidly changing, so too are prevention and identification techniques. The use and publication of the checklist in Multimedia Appendix 1 of this paper can lead to more transparency around the experiences of different studies. This may allow for future exploration of variation in fraudulent rates, and possible reasons for these (eg, platform, incentive, or recruitment method). Purpose-designed empirical studies are needed to determine the most effective platform-, methodology-, and population group–specific strategies for preventing and identifying bot or scam completions.

### Conclusions

Researchers using online recruitment and data collection methods should consider the checklist of strategies provided to reduce and identify potential bot or scam completions at each stage of their project including in the creation of study collateral, survey design and development, data review, and write-up. Continued exploration and sharing of strategies are needed as technology and bots or scams evolve. Details of the steps planned or taken should be included in grant and ethics applications. While these steps may be time- and resource-consuming, they may help prevent and identify potentially nonlegitimate survey responses and provide greater confidence in the research findings.

## Data Availability

The datasets generated or analyzed during this study are not publicly available due to ethical approval not permitting this, but are available from the corresponding author on reasonable request.

## Appendix

Multimedia Appendix 1 Checklist to reduce the incidence of, and identify potential fraudulent completions of online surveys in health research [Arundell L, Salmon J, Walsh A, et al. Let’s Talk aBOT Scam Online Survey Completions in Health Behavior Research: A Tutorial with Case Studies, Practical Guidance, and a Checklist for Researchers. JMIR Public Health Surveill. 2025;11:e76622. doi: 10.2196/76622].

## Abbreviations

REDCap: Research Electronic Data Capture
