# Supplementary material for: Let’s Talk aBOT Scam Online Survey Completions in Health Behavior Research: Tutorial With Case Studies, Practical Guidance, and a Checklist for Researchers
Source: JMIR Public Health Surveill. 2025 Dec 24;11:e76622. doi: 10.2196/76622 (PMC12780700; doi:10.2196/76622)
Supplement: Multimedia Appendix 1 [file publichealth_v11i1e76622_app1.pdf]

Multimedia Appendix 1: Checklist to reduce the incidence of, and identify potential fraudulent completions of online surveys in health research

**Research phase**	**Strategies to reduce incidence of, and to identify, potentially fraudulent completions**	**Strategy used**<br><br>This assists the planning and conduct of studies	**Number (%) of potential fraudulent completions identified**<br>This assists the analysis and write up of findings.
i. Before data collection: Creating study collateral	• Limit the prominence of advertising vouchers or payment as incentives	☐Yes ☐No ☐NA	
	• Specify the type of voucher and where it can be redeemed (eg, for an Australian retailer)	☐Yes ☐No ☐NA	
	• Specify that completions are limited to one per household	☐Yes ☐No ☐NA	
	• Specify that suspected fraudulent completions will not receive the voucher	☐Yes ☐No ☐NA	
	• Additional project specific strategies (describe):	☐Yes ☐No ☐NA	
ii. Survey design and development I: enable all available in-built survey platform functions	Platform specific but may include:		
	• reCAPTCHA	☐Yes ☐No ☐NA	
	• Prevention of multiple submissions	☐Yes ☐No ☐NA	
	• IP address or geolocation tracking	☐Yes ☐No ☐NA	
	• Fraud or scam or bot identification scores	☐Yes ☐No ☐NA	
	• Additional project specific strategies (describe)	☐Yes ☐No ☐NA	
iii. Survey design and development II: online survey question and structure	• Include reCAPTCHA variable	☐Yes ☐No ☐NA	
	• Include branching logic to exclude ineligible responses from progressing	☐Yes ☐No ☐NA	
	• Include two-step process separating screening from consent form completion, with use of individualised or personal links following screening	☐Yes ☐No ☐NA	
	• Include automated date and time survey fields	☐Yes ☐No ☐NA	
	• Include repeat questions where the response would not typically change	☐Yes ☐No ☐NA	
	• Include dummy 'honeypot' questions	☐Yes ☐No ☐NA	
	• Include attention check questions to progress	☐Yes ☐No ☐NA	
	• Request additional contact information to verify participant eligibility	☐Yes ☐No ☐NA	
	• Additional project specific strategies (describe):	☐Yes ☐No ☐NA	
iv. Following data collection: identifying indicators of potential bot or scam completions	• Completed 'honeypot question' question	☐Yes ☐No ☐NA	
	• Incorrect geolocation for the study sample	☐Yes ☐No ☐NA	
	• Inbuilt bot detection function or variables suggest fraud	☐Yes ☐No ☐NA	
	• Duplicate IP addresses	☐Yes ☐No ☐NA	
	• Near identical IP addresses	☐Yes ☐No ☐NA	
	• Failed a reCAPTCHA test, including several failed attempts at passing, followed by a successful completion from the same IP address	☐Yes ☐No ☐NA	

	• Unrealistic survey completion time (include mean and cut-off used)	☐Yes	☐No	☐NA	
	• Multiple consecutive responses with similar survey start or end time, or in a run of start or end times	☐Yes	☐No	☐NA	
	• Multiple participants with the same personal details	☐Yes	☐No	☐NA	
	• Questionable email address or names	☐Yes	☐No	☐NA	
	• Unusual responses for study context	☐Yes	☐No	☐NA	
	• Mismatch between data provided for the same question at different points of the enrolment or completion process	☐Yes	☐No	☐NA	
	• Identical or very similar responses to an open-ended question	☐Yes	☐No	☐NA	
	• Questionable date format used for date-based responses	☐Yes	☐No	☐NA	
	• Landline phone number provided when a mobile number is requested, or provided landline phone numbers do not match the area or city they are claiming to live in	☐Yes	☐No	☐NA	
	• Full name provided when only a first name was requested	☐Yes	☐No	☐NA	
	• Non-response to requested contact information	☐Yes	☐No	☐NA	
	• Completed at unusual or unlikely times for study population	☐Yes	☐No	☐NA	
	• Additional project specific data checks (describe):	☐Yes	☐No	☐NA	
v. Analysis and write-up: reporting of potential bot or scam completions	• Describe steps to avoid potential fraudulent completions	☐Yes	☐No	☐NA	
	• Include a description of how potential fraudulent completions were identified	☐Yes	☐No	☐NA	
	• Include the number of fraudulent completions identified	☐Yes	☐No	☐NA	
	• Confirm that fraudulent completions were excluded	☐Yes	☐No	☐NA	
	• Additional project specific reporting (describe):	☐Yes	☐No	☐NA	

NA: not applicable

Reference: Arundell L, Salmon J, Walsh A, et al. Let’s Talk aBOT Scam Online Survey Completions in Health Behavior Research: Tutorial with Case Studies, Practical Guidance, and a Checklist for Researchers. JMIR Public Health Surveill. 2025;11:e76622. [doi: 10.2196/76622]
